# Chemo-Mechanical Characteristics of Mud Formed from Environmental Dust Particles in Humid Ambient Air

**DOI:** 10.1038/srep30253

**Published:** 2016-07-22

**Authors:** Ghassan Hassan, B. S. Yilbas, Syed A. M. Said, N. Al-Aqeeli, Asif Matin

**Affiliations:** 1 Department of Mechanical Engineering, King Fahd University of Petroleum and Minerals (KFUPM), Dhahran 31261, Saudi Arabia; 2 Center of Research Excellence in Renewable Energy (CoRE-RE), King Fahd University of Petroleum and Minerals (KFUPM), Dhahran 31261, Saudi Arabia; 3 Center of Engineering Research, King Fahd University of Petroleum and Minerals (KFUPM), Dhahran 31261, Saudi Arabia

## Abstract

Mud formed from environmental dust particles in humid ambient air significantly influences the performance of solar harvesting devices. This study examines the characterization of environmental dust particles and the chemo-mechanics of dry mud formed from dust particles. Analytical tools, including scanning electron microscopy, atomic force microscopy, energy dispersive spectroscopy, particle sizing, and X-ray diffraction, are used to characterize dry mud and dust particles. A micro/nano tribometer is used to measure the tangential force and friction coefficient while tensile tests are carried out to assess the binding forces of dry mud pellets. After dry mud is removed, mud residuals on the glass surface are examined and the optical transmittance of the glass is measured. Dust particles include alkaline compounds, which dissolve in water condensate and form a mud solution with high pH (pH = 7.5). The mud solution forms a thin liquid film at the interface of dust particles and surface. Crystals form as the mud solution dries, thus, increasing the adhesion work required to remove dry mud from the surface. Optical transmittance of the glass is reduced after dry mud is removed due to the dry mud residue on the surface.

Recent changes in climate have resulted in severe and frequent dust storms around the globe, particularly in the Middle East and North Africa (MENA) region. Dust settling onto surfaces causes irreversible damage to the surfaces and lowers the system performance, such as the efficiency of solar thermal and solar photovoltaic (PV) systems. Many surface treatment methods have been reported to minimize the effect of dust particles on the optical characteristics of glass covers or reflective surfaces^1^,^2^. However, the self-cleaning or cost-effective removal of dust particles from such surfaces still remains challenging. In humid air environments, water vapor condensates on the surface of dust particles and is then absorbed by particles forming mud on the solid surface. The formed mud dries as a result of solar radiation and adheres to the solid surface. Dry mud removal from surfaces becomes difficult due to the strong adhesion between the dry mud and surface. Although previous studies have focused on the adhesion of particles on surfaces^3^,^4^, the chemo-mechanics of mud adhesion still requires further analysis.

Numerous studies have examined environmental dust and its effects on glass covers and reflective surfaces. Mekhilef *et al*.^5^ reviewed the effects of humidity level, dust accumulation, and wind velocity on the performance of PV panels. They demonstrated that the humidity, dust accumulation, and the speed affected the PV panel performance to similar extents. Mani and Pillai^6^ discussed dust accumulation and its effect on the performance of a PV module. These authors introduced a maintenance/cleaning cycle for PV systems that accounts for the prevalent environmental and climatic conditions, which are mainly dust accumulation. The authors further demonstrated that dust deposition and accumulation had a significant effect on the performance of solar panels. Rajput *et al*.^7^ studied the influence of dust deposition on the electrical efficiency of monocrystalline PV modules. Their findings revealed that the dust accumulation reduced the maximum device efficiency by 90%. Ghazi *et al*.^8^ presented a review on dust accumulation on flat surfaces in the MENA region. These researchers found that the MENA region exhibited the highest amount of dust deposition in the world. Sudan had the highest dust deposition rate (9 times greater than that in the United Kingdom). Boyle *et al*.^9^ investigated the influence of dust accumulation on the optical properties of the glass covers of PV panels in the USA. They demonstrated that the incidence angle of irradiance varied linearly with dust accumulation and that the transmittance of the glass cover was affected by the local accumulation of dust particles. Said and Walawil^10^ investigated the influence of dust accumulation on the transmittance of a PV module cover glass and found that dust accumulation had a detrimental effect on PV panel performance; specifically, the power output was reduced by 6% for dusty PV panels, and the short circuit current was reduced by 13% after one month of exposure to the environment. Said *et al*.^11^ investigated the possibility of utilizing antireflective coatings and textured glass to reduce dust fouling and found that textured and coated surfaces reduced the effect of dust accumulation on the cover glass; in contrast, texturing the surfaces of PV panels increased the module temperature, which affected the power output. Rahman *et al*.^12^ performed an experimental study assessing the effects of relative humidity, ambient temperature and dust accumulation on PV panel output and found that the device output power decreased significantly when the relative humidity increased by 20% with a dust sedimentation of 0.012 g/cm^2^. The wind speed has a significant effect on dust movement and accumulation on surfaces. Wind causes the spreading and transferring of dust particles within the atmosphere, which might increase deposition layers. As the wind speed increases, a large amount of dust particles move in the air and dust particle sedimentation decreases on Earth’s surfaces, which results in the deterioration of a solar cell’s fill factor^5^. However, in some situations, the wind stream could blow the dust particles away from PV panel surfaces, which could decrease dust accumulation^13^. Similar findings were reported by Hegazy^14^, who found that dust accumulation was significantly reduced on windy days and that this reduction was more pronounced as the panel tilt angle was increased.

Mekhilef *et al*.^5^ reported that adhesion of dust particles to surfaces was affected by the humidity in the atmosphere. As the relative humidity decreased, the solar panel efficiency increased because less dust adhered to the surface. In addition, Adinoyi and Said^15^ demonstrated that dust particles adhered to the surface of the PV panel cover glass due to humidity, which required external efforts to carefully clean the surfaces to restore the initial power output of the panels. Brown *et al*.^4^ reported that applying an anti-soiling hydrophilic coating to the glass cover reduced the amount of dust soiling on the surface. Conversely, capillary bridges formed on the solid surfaces because of the interaction between the dust particles and condensed vapor in the gaps between the particles and surface. This effect generated meniscus forces that increased the dust layer and the adhesion force between the dust particles and solid surfaces^16^,^17^. Corn^18^ studied the adhesion force of solid particles and demonstrated that the adhesion force increased with the particle size. Furthermore, the contact area between a rough surface and particle was found to have a major role in the adhesion between the particles and surface. In addition, the relative humidity of the ambient air affected the adhesion force. McLean^19^ presented the cohesive forces related to the sediment layers of dust particles. He demonstrated that an electrostatic precipitator had a significant cohesive force that influenced the sediment layers because of the electric field charging of the particles. Podczeck *et al*.^20^ studied the effect of the relative humidity on particle adhesion and found that at high relative ambient humidity, the adhesion increased slightly, whereas the van der Waals forces became nearly 10 times greater than the electrostatic forces. Somasundaran *et al*.^21^ examined the adhesion force between solutions on glass surfaces and found that cohesive forces on the glass surface increased when the pH of the solution decreased; this effect was associated with the amount of salt in the solution. In addition, the cohesion between particles and surfaces was reduced due to the interaction of an anionic surfactant within the polyethylene oxide layer. Fukunishi and Mori^22^ investigated the adhesion force between particles in humid environments and demonstrated that the adhesive force between hydrophobic glass and the particles remained nearly constant for different humidity conditions. Kumar *et al*.^23^ studied the influence of particle size on the adhesion of particles to smooth surfaces and used the Johnson-Kendall-Roberts (JRK) adhesion model to characterize the adhesion force for smooth surfaces. Jarząbek *et al*.^24^ presented a measurement method to determine the particle adhesion of ceramic and the adhesion in ceramic-reinforced composite structures. They demonstrated that the presence of ceramics improved the adhesion of particles in the composite matrix. Knoll et al.^25^ characterized the adhesion force generated by magnetic particles on protein surfaces. Their findings revealed that magnetic particles strongly adhered to protein surfaces and that an additional force was required for the separation of magnetic particles. Petean and Aguiar^26^ determined the adhesive force between a particle and rough surface and compared experimental data with the simulation results of various models. Their findings indicated that among the models considered, the JKR model yielded results that were closest to the experimental values.

Yilbas *et al*.^27^ reported that dust particles consist of ionic and neutral compounds. The alkaline and alkaline earth metallic compounds of dust particles dissolve in the water condensate on surfaces in humid environments, which gives rise to the formation of a chemically active mud solution that flows around dust particles under the effect of gravity and reaches the solid surface where the dust particles have settled. This, in turn, gives rise to the formation of a liquid film between the dust particles and solid surface. The liquid film dries together with the mud, thus forming a dry mud on the solid surface. However, the bonding force is a combination of the ionic and adhesion forces that depends on the wetting area of the solid surface and the dust particles. Although research studies have reported adhesion between particles and surfaces^23^,^28^, no studies have reported on the influence of a dry mud solution formed at the interface on adhesion. Therefore, the present study investigates bonding among dust particles in dry mud. Adhesion and cohesion forces are determined experimentally using micro/nano tensile tests. The adhesion of the dust particles and mud residues on glass surfaces is also determined using scratching tests. Analytical tools, including scanning electron microscopy (SEM), atomic force microscopy (AFM), energy dispersive spectroscopy (EDS), and X-ray diffraction (XRD), are used to characterize the dust particles prior to mud formation.

## Experimental

Optical glass with a thickness of 1 mm and excellent optical clarity was used as the workpieces to experimentally determine the adhesion and cohesion forces of dry mud particles on the surface. The glass was ultrasonically cleaned prior to mud formation from environmental dust and water mimicking water condensation on dust particles. Although the deposition rate of dust particles over the Kingdom of Saudi Arabia varies with seasons^29^,^30^, the chemical composition of dust particles remains almost same in various locations of the Kingdom^31^. This is mainly because of the localized wind effects on desert environment, where dust particles migrate and form dust storms in the Kingdom^32^. Consequently, in the present study, dust analysis is concentrated in the northern region of Saudi Arabia where air humidity remains high because of close location to the Arabian Gulf. Dust particles were collected from the local environment in the Dammam area of Saudi Arabia, and a 300-μm-thick layer of dust particles was formed on the glass surfaces. A dust layer with a thickness of 300 μm resembles actual dust accumulation on surfaces in an open environment after a one-week period during regular sand storms. Desalinated water having the same volume as the dust layer was gradually dispensed onto the dust layer on the glass surface in a temperature-controlled cell. Certain tests were initially carried out to measure the amount of water vapor absorbed by dust particles due to condensation in the local humid environment over a 6 h period. The tests results revealed that the amount of condensate had almost the same volume as the dust after 6 h. Dispensed water with the same volume as the dust particles was applied to the glass surface without mechanical mixing in a temperature-controlled cell to simulate water condensation on the layer of dust particles in humid air. This process gave rise to natural mud formation at the workpiece surfaces at room temperature. The duration of mud drying was 36 h from the start of the drying process. Once the mud was dried, the glass surface with the dry mud was measured to determine the adhesion work and friction coefficient. Upon completion of the adhesion and friction tests, the dry mud layer was removed from the glass surface using pressurized desalinated water jet with a diameter of 2 mm and a velocity of 2 m/s. The water jet-assisted cleaning process was conducted 20 min for each workpiece surface. Finally, analytical tools were used to assess the morphology and optical transmittance characteristics of the cleaned glass surfaces.

We used analytical tools to characterize the dry mud and glass surface. Scanning electron microscope-energy dispersive X-ray spectrometer (SEM-EDX) analyses were performed using a Jeol 6460 electron microscope, Massachusetts, USA. SEM-EDX resolution mode ranged 3.0 nm (30 kV), 8 nm (3 kV) and 15 nm (1 kV), and maximum magnification was ×300,000. XRD analysis was carried out using a Bruker D8 Advanced with CuKα radiation. The typical XRD settings were 40 kV and 30 mA with a scanning angle (2θ) that ranged from 20° to 90°. The surface texture was analyzed using AFM/SPM microscopy (Agilent) operating in contact mode. The AFM microscope tip was made of silicon nitride (*r* = 20–60 nm) with a manufacturer-specified force constant, *k*, of 0.12 N/m.

The friction coefficient and tangential force for the adhesion work calculations were measured using a linear micro-scratch tester (MCTX-S/N: 01-04300). During the experiments, the equipment was set at a contact load of 0.03 N and an end load of 5 N. The total length for the scratch tests was 10 mm, and the scanning speed was maintained at 5 mm/min with a loading rate of 5 N/s.

**Figure 1:**
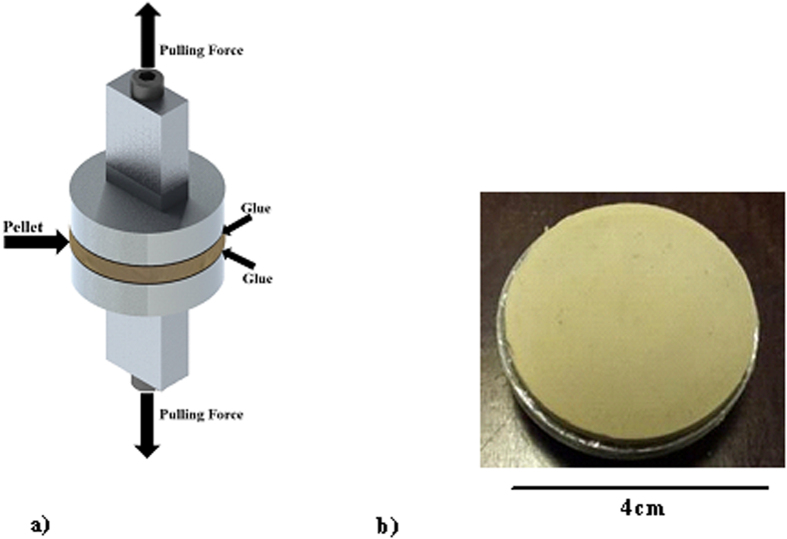
Images of the fixture and dry mud pellet prepared for the tensile tests. (**a**) fixture used in the tensile tests and (**b**) optical image of a dry mud pellet.

Circular mud pellets were formed in a manner similar to the procedure adopted to form the mud on the glass surfaces. Mud pellets were dried for 72 h prior to tensile tests. A typical optical image of a mud pellet is shown in Fig. 1. The size of the mud pellets (4 cm-diameter and 5 mm-thick pellets) was fixed according to fixture designed to hold the samples within the tensile test machine. Both dried mud pellet surfaces were glued to the fixture surfaces using a strong adhesive (3 M Scotch-Weld) and placed into the tensile machine sample holders. Micro/nano tensile equipment (BOSE, Model: 3220) was operated using a constant displacement rate of 0.005 mm/s with a maximum load of 200 N and a maximum displacement of 6.5 mm.

## Results and Discussion

The characteristics of the environmental dust and mud formed from the dust particles were examined. The adhesion between the dust particles in the dry mud was assessed using analytical tools. In addition, the adhesion between the dry mud residue and glass surface was assessed using micro/nano scratch tests. The findings are presented below.

### Characterization of Dust Particles

**Figure 2:**
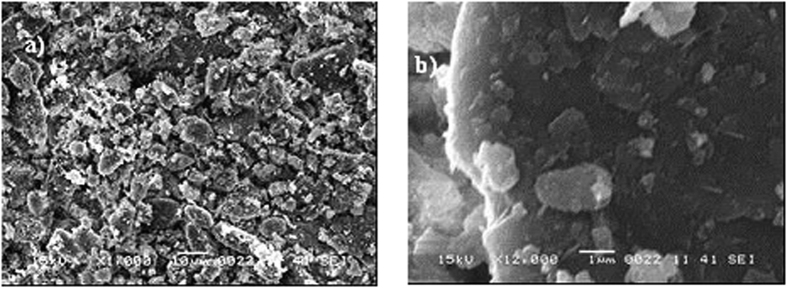
SEM micrograph of dust particles. (**a**) various sizes of dust particles and (**b**) small dust particles adhering to the surface of large dust particles.

**Figure 3:**
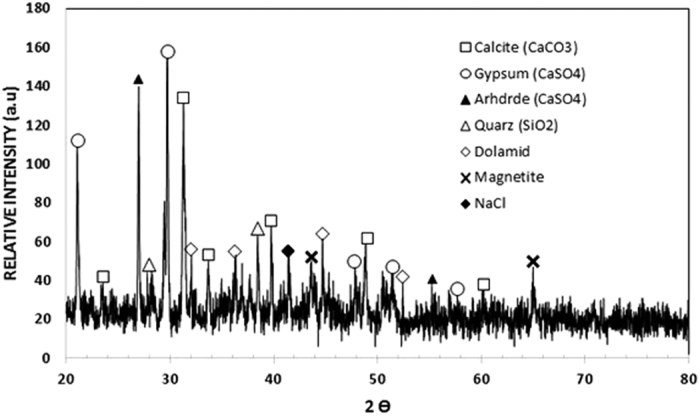
X-ray diffractogram for the dust particles.

Figure (2) shows SEM micrographs of the dust particles. In general, the dust particles were composed of various sizes and shapes. Small dust particles adhere to large particles in a manner determined by the electrostatic charges of the small particles. Small dust particles were exposed to solar radiation for long durations, and the attachment of ionic compounds to these particles in regions close to the sea causes static charging of these particles^33^. The shape of the dust particles can be categorized by two key geometric parameters: the shape factor (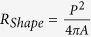, where *P* is the perimeter of the dust particle) and the aspect ratio (
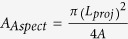
, where *A* is the cross-sectional area and *L*_*proj*_ is the longest projection length of the dust). The shape factor is related to the inverse of the particle circularity in relation to the complexity of the particle; in which case, a shape factor of unity corresponds to a perfect circle. The aspect ratio corresponds to the ratio of the major axis to the minor axis of the ellipsoid best fit to the particle, which is associated with the approximate particle roundness. Consequently, the equivalent circular area for the round shapes can be determined from the measured particle sizes. In the case of non-circular shapes, an ellipse is considered by assuming that the longest projection is the major axis and that the particle cross-sectional area is preserved. The relationship between the particle size and the aspect ratio or the shape factor is not simple. However, a simplified assessment can be introduced to classify dust particles in terms of their shapes. Because an inverse relationship is observed between the particle size and aspect ratio, an increase in the particle sizes results in low aspect ratios. However, there is a direct relationship between the particle size and shape factor. Consequently, the shape factor increases and the aspect radio decreases with increasing particle size. The shape factor becomes approximately 1 for small particles (≤2 μm), and the median shape factor approaches 3 for large particles (≥10 μm). Although the particle size varies from the sub-micron scale to tens of microns, the average particle size is on the order of 1.2 μm. The EDS data (wt%) of the dust particles are provided in Table 1. Dust particles contain various elements, such as Si, Ca, Mg, Na, K, Cl, S, O and Fe. The presence of oxygen in the EDS data indicates that the particles include the oxide compounds, and the presence of Cl reveals the presence of salt compounds in the dust particles. This result is attributed to the prolonged duration that dust remains in the atmosphere close to the Arabian Gulf. The dust particles were collected in Dhahran city, which is close to the Arabian Gulf. Figure 3, which provides the X-ray diffractogram for the dust particles, also indicates that the dust particles contain salt and oxide compounds. The peaks corresponding to K, Na, Ca, S, Cl, and Fe are indicated in the diffractogram. The peaks for Na and K are probably related to salt from the Arabian Gulf. Sulfur can be associated with calcium, such as in anhydrite or gypsum components (CaSO_4_), in dust particles. Iron is likely related to clay-aggregated hematite (Fe_2_O_3_).

### Characterization of Mud Solution and Dry Mud

**Figure 4:**
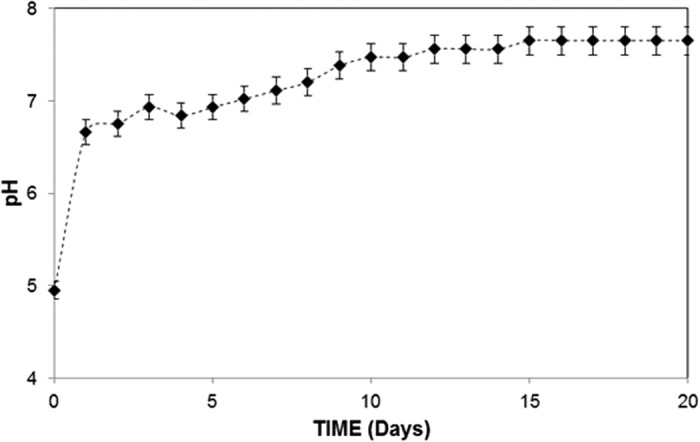
Temporal variation of the pH of the mud solution.

**Figure 5:**
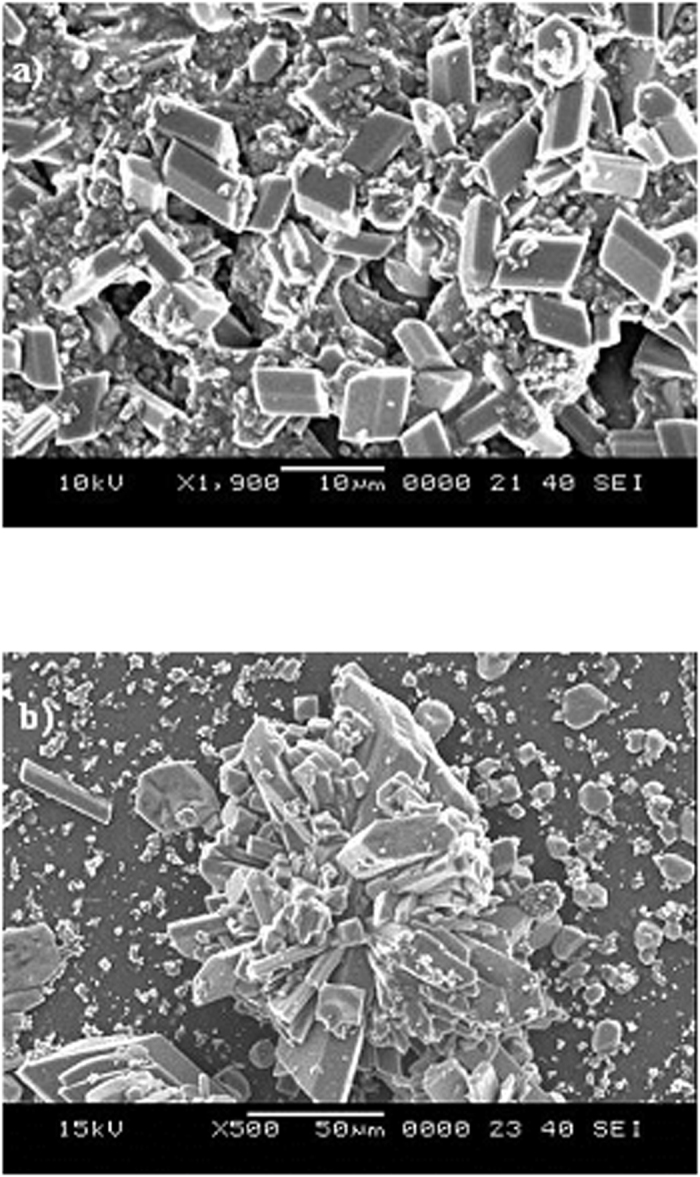
Scanning electron micrograph of crystalized structures formed on the glass surface after the mud solution dried.

The mud solution (liquid) was extracted from the mixture of dust particles and water. The pH of the mud liquid was measured and analyzed using inductively coupled plasma mass spectroscopy. The temporal variation of the mud liquid pH is shown in Fig. 4, and the data obtained from the inductively coupled plasma mass analysis are shown in Table 2. The pH of the mud liquid increased significantly with time and achieved an approximate steady state after 10 days. The pH of the solution is basic in nature, and the high rate of increase in the pH is associated with the presence of OH^−^ ions in the mud solution, which is related to the dissolution of alkaline (Na, K) and alkaline earth (Ca) metals in the dust particles. This trend can also be observed from Table 1, where alkaline and alkaline earth metals are present. To assess the characteristics of the dry mud solution, mud liquid was placed on a glass surface and dried in a controlled environment (at 20 °C and a pressure of 1 atm). Figure 5 shows the SEM micrographs of the dried mud solution on the glass surface. Various crystal structures sizes were formed on the glass surface (Fig. 5a,b). Although drying took place in a controlled environment, the local heat transfer modifies the crystal size on the glass surface^34^. In this case, the crystal structures become small when the local cooling rates are high at the surface (Fig. 5a), whereas the crystal structures are large under low cooling rates (Fig. 5b)^34^. The elemental composition of the crystallized structures on the glass surfaces was analyzed, and Table 3 provides the relevant EDS data. The crystallized layer shows that alkaline (K, Na), alkaline earth metals (Ca), oxygen and chlorine were present in the crystal structures. Therefore, the alkaline and alkaline earth metal compounds dissolve in water and form a chemically active liquid at the glass surface. The mud solution has chemically active characteristics^35^. However, assessing the chemical potential of the mud solution is not within the scope of this study and will be studied in the future.

**Figure 6:**
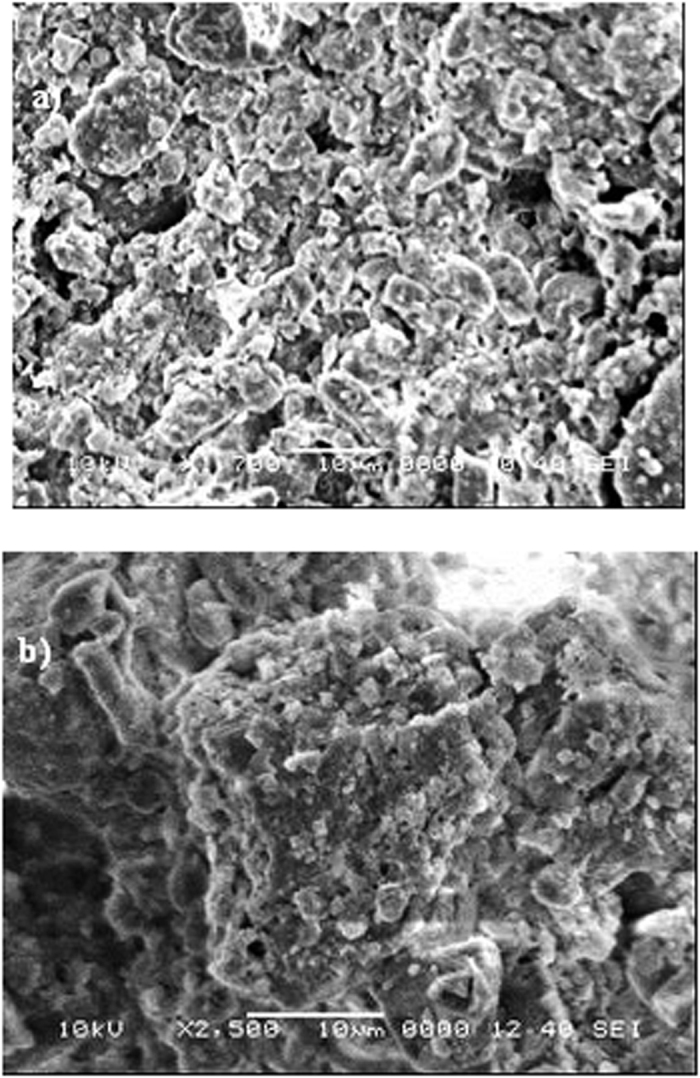
Scanning electron micrograph of the mud surface. (**a**) voids formed around large dust particles, and (**b**) dense structures formed around small dust particles owing to the mud solution.

**Figure 7:**
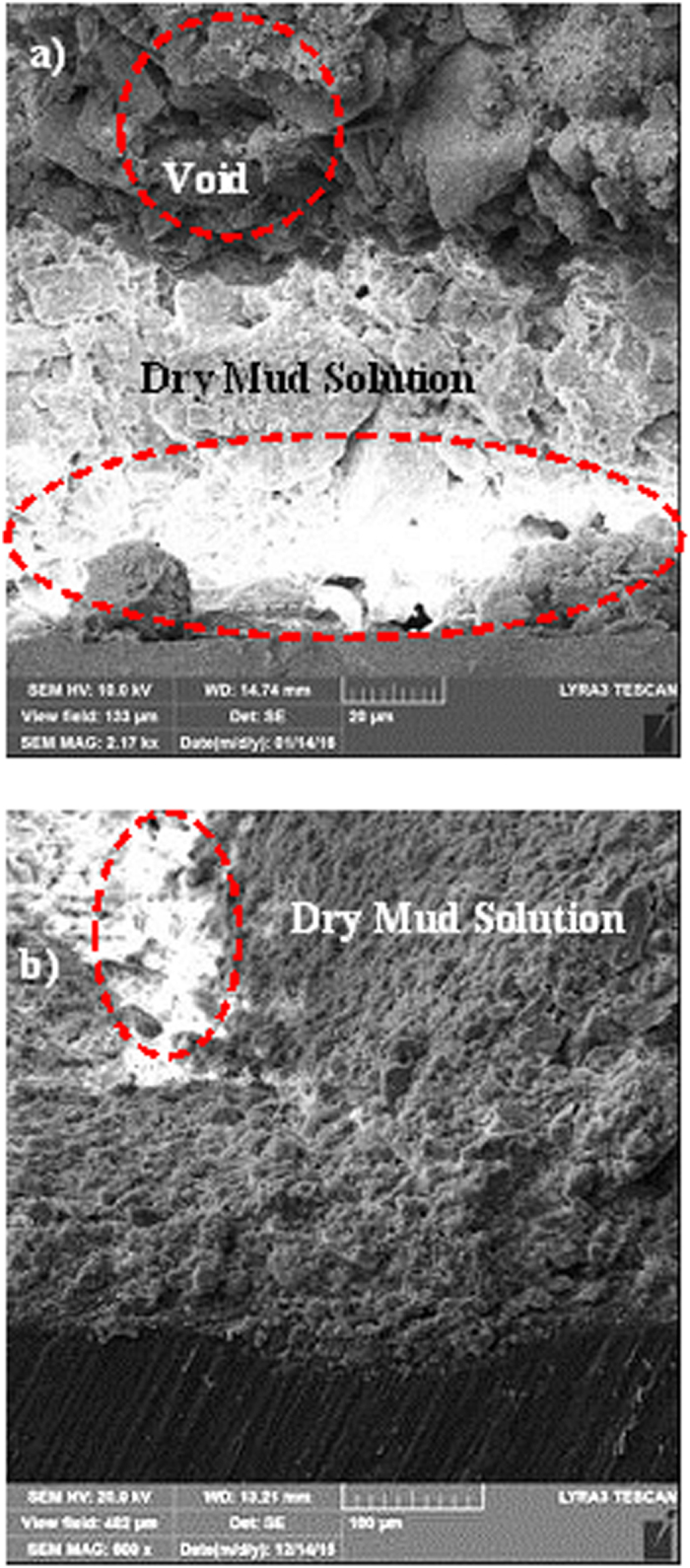
Scanning electron micrograph of the dry mud cross-section. (**a**) dry mud solution at the dry mud-glass surface interface and (**b**) dry mud solution in a void.

**Figure 8:**
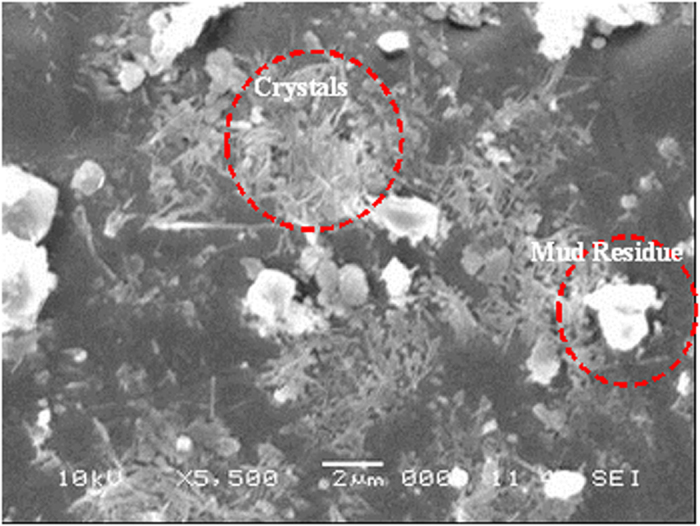
Scanning electron micrograph of the glass surface in which the dry mud was removed using a pressurized desalinated water jet.

**Figure 9:**
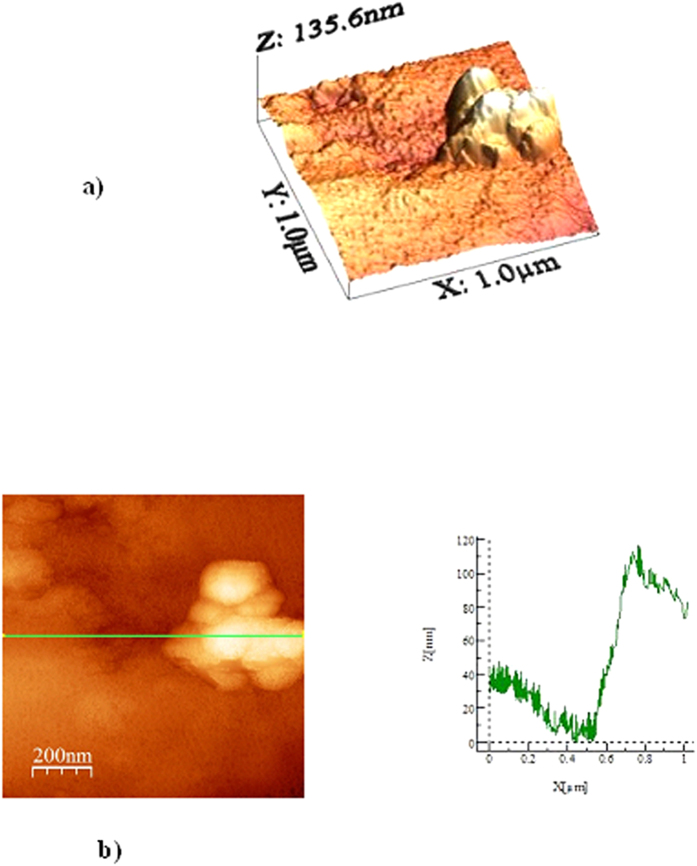
Atomic force micro-images of the glass surface after the dry mud was removed using a pressurized desalinated water jet. (**a**) 3-D image of the surface and (**b**) a line scan across the surface and the texture height.

Figures 6 and 7 show SEM micrographs of the dry mud surface and dry mud cross-section, respectively. Large particles together with closely spaced small particles were observed on the surface of the dry mud (Fig. 6a) whereas voids were observed near certain large particles (Fig. 6b). In addition, some small voids occurred on the dry mud surface; these voids are mainly associated with the evaporation of water from the mud surface during drying, which causes the porous-like morphology at the surface. However, mud contains alkali and alkali earth metal compounds (Table 3), which dissolve in water during the formation of the mud solution and flow across the mud cross-section toward the glass surface as a result of gravity. The mud solution accumulates on the glass surface and forms a thin liquid layer. Upon drying, a crystallized dry mud solution is formed between the dry mud and glass surface. This situation can be observed in Fig. 7a, in which the SEM micrographs of the dry mud cross-section are shown. The dry mud cross-section consists of porous-like structures, including some small cavities across the cross-section (Fig. 7b) because of the wide range of dust particle sizes (0.001–20 μm). This arrangement enables the liquid mud solution to flow in between these structures. However, a portion of the liquid mud solution sediments in the cavities across the cross-section (Fig. 7a). This sedimentation appears as a bright color in the SEM image (Fig. 7b). To assess the effect of dry mud and its solution on the glass surface, the dry mud was removed from the glass surface using a pressurized distilled water jet. Figure 8 shows SEM micrographs of the dust particle residue on the glass surface. The residue of the dry mud was related to the dry mud solution in between dust particles and the glass surface, which increased the adhesion at the interface. The EDS analysis of the dry mud residue revealed that the dry mud residue was composed of Ca and Si, and Na, K and Cl were also present (Table 4). These results indicate that the residue of the dry mud solution together with dry mud particles were present on the glass surface (Fig. 8). In addition, close examination of the SEM micrograph (Fig. 8) reveals that some crystallized structures were formed on the surface; in this case, dissolved alkaline and earth alkaline metals are responsible for the crystalline morphology on the dry mud that was removed from the glass surface. Figure 9 shows an AFM image of the dry mud removed from the glass surface. Dry mud residue formed various texture morphologies on the glass surface, which is evident in the 3-dimensional image of the surface shown in Fig. 9a. The texture height at the surface due to mud residue varies, as can be observed from the line scan of the dry mud removed from the surface (Fig. 9b). The maximum height varies within a range of 130 nm; therefore, the average surface roughness of the glass surface increases to 80 nm. In addition, some small cavities formed at the glass surface after the dry mud was removed. The formation of small cavities is associated with hydroxyl attached to the surface because of the high pH of the liquid mud solution prior to drying^27^. Consequently, dry mud residues modify the surface texture and surface structure of the glass through hydroxyl attacks on the surface.

### Mechanical Properties of Dry Mud

The mechanical properties of dry mud were assessed through friction and tensile tests. The tangential force was monitored during the friction tests to determine the adhesion work required to remove dry mud from the glass surface. Tensile tests provide a combination of the adhesion and cohesion forces (binding forces) in the dry mud; therefore, the cohesion forces in dry mud caused by drying were also assessed by comparing the tangential force and tensile test data.

**Figure 10:**
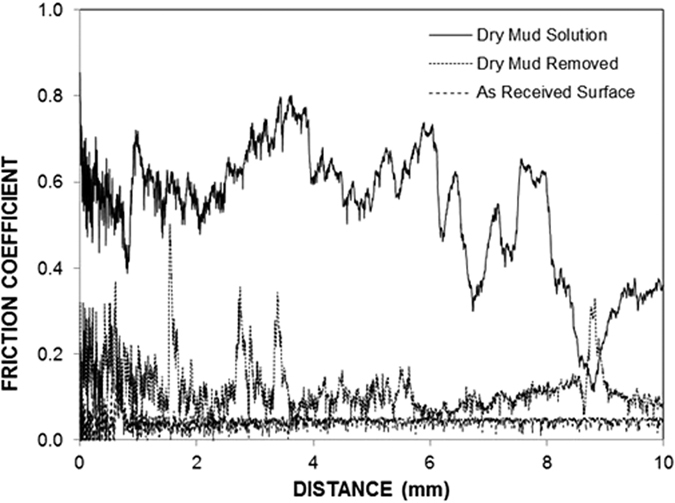
Friction coefficient for the dry mud solution, as-received glass surface, and surface for which the dry mud was removed using a pressurized desalinated water jet.

**Figure 11:**
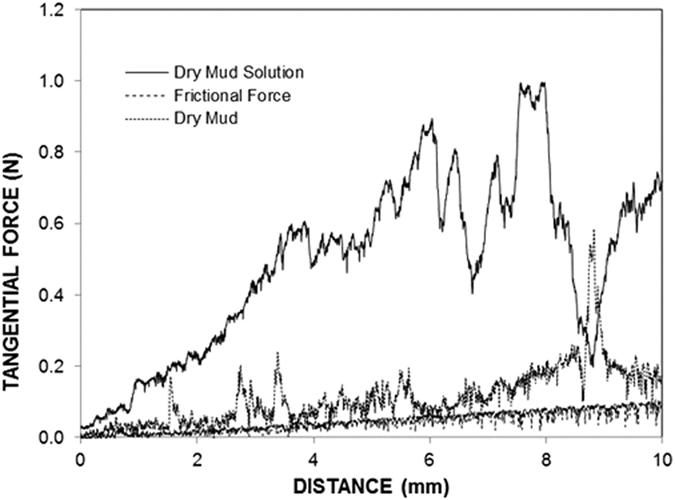
Tangential force obtained from the scratch tests for the dry mud solution and surface for which the dry mud was removed using a pressurized desalinated water jet. The frictional force for the as-received glass surface is provided for comparison.

Figure 10 shows the friction coefficients of the as-received surface, dry mud solution and dry mud removed using a pressurized distilled water jet. Figure 11 shows the tangential forces obtained when the dry mud was removed from the glass surface, the frictional force of the glass surface without mud deposition and the tangential force corresponding to the dry mud solution being removed from the glass surface. The area under the force curves provides the frictional and adhesion work. The friction coefficient is largest for the dry mud solution, followed by the surface after the dry mud was removed using the pressurized distilled water jet and then as-received surface. The friction coefficient increases due to the strong adhesion between the indenter tip and dry mud solution surface, as shown in Fig. 11. Moreover, the increased friction coefficient for the surface for which the dry mud has been removed is associated with an increased surface roughness because of the mud residues on the surface. Increasing the surface roughness has been reported to increase the friction coefficient of the surface^36^. In addition, some small peaks in the friction curve are observed, which is related to the surface roughness due to the mud residue on the surface. In addition, a locally increasing friction coefficient is also related to the surface modification because of the hydroxyl attacks on the surface as the mud solution dries on the glass surface. Consequently, the increase in surface roughness due to the mud residue and the cavities formed because of the hydroxyl attacks contribute to the enhancement of the friction coefficient of the surface. The scratch marks left on the as-received glass surface and the surface for which the dry mud has been removed by a pressurized distilled water jet extend nearly uniformly on the surface. However, a portion of the mud residue on the surface modifies the scratch marks owing to the strong adhesion force between the mud and glass surface because of the thin film formed from the dried mud solution at the interface. Moreover, no micro-cracks were observed around the scar marks in the as-received glass and in the glass in which the mud was removed. This result demonstrates that the reduction in the fracture toughness due to surface modification by hydroxyl attack is not significant. In the case of the tangential force (Fig. 11), a strong adhesion between the dry mud and glass surface resulted in a sharp increase in the tangential force. This appears locally on the force curve (Fig. 11). Consequently, the dry mud solution between the dry mud and glass surface is responsible for the increase in tangential force. Table 5 provides the adhesion work determined from the tangential force measured using the scratch tester during dry mud removal. The adhesion work is obtained by integrating the tangential force over the scratch distance. However, the adhesion work determined is corrected by subtracting the frictional work, which was obtained by integrating the frictional force over the scratch distance for the as-received glass surface. The adhesion work determined was on the order of 0.119 mJ. The experiments for the tangential force variation were repeated five times to assess any experimental error. The experimental error was on the order of 17% based on the repeatability tests.

**Figure 12:**
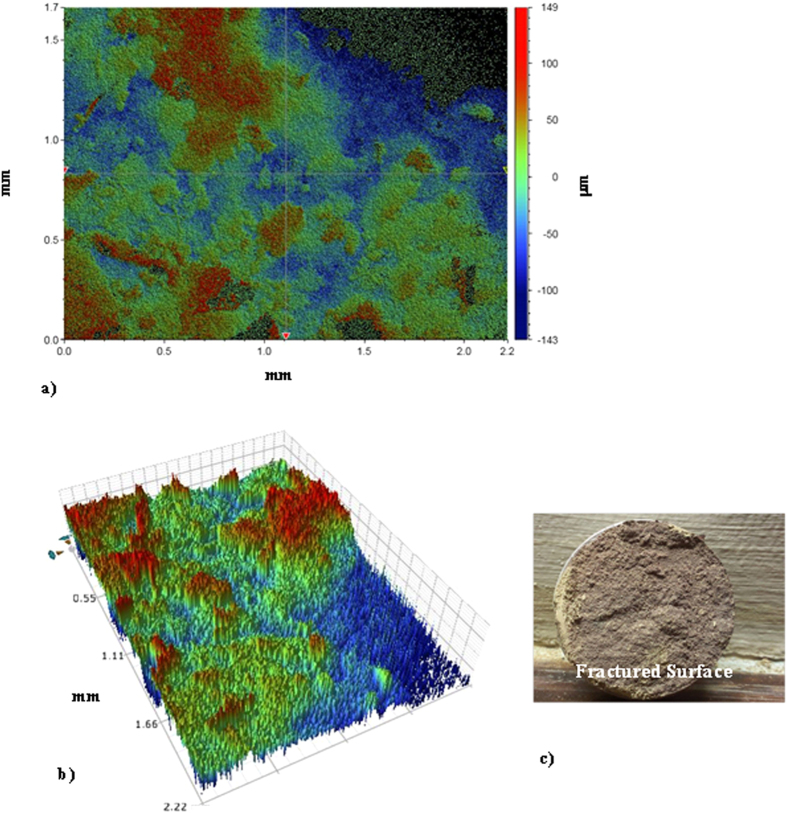
Optical image of the fractured pellet surface after tensile tests . (**a**) 2-dimensional image showing the texture height distribution, (**b**) 3-dimensional view of the texture of a fractured surface, and (**c**) optical image of the fractured surface (pellet diameter is 40 mm).

**Figure 13:**
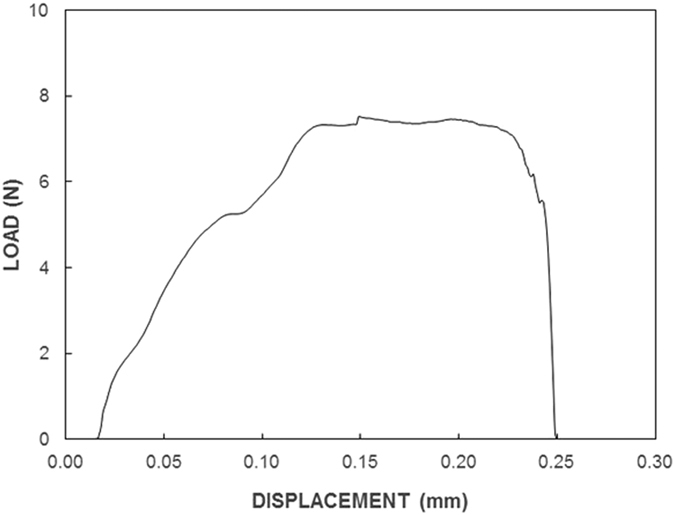
Load-displacement curve for the tensile test of a dry mud pellet.

Figure (12) shows optical images of the dry mud pellet after the tensile tests. Figure 12a is a zoomed-in two-dimensional image of the fractured surface, Fig. 12b is the three-dimensional images of the fractured surface; and Fig. 12c presents the fractured pellet. Figure 12 shows the pulling force with the displacement obtained from the tensile tests. In Fig. 13, the pulling force increases to reach a maximum without going through a yielding point, as opposed to the force observed for dense materials, such as metals. In addition, no necking was observed in the tensile curve (Fig. 13). Necking is a mode of tensile deformation in which relatively large amounts of strain localize disproportionately in a small region of the material; in this case; the resulting decrease in the local cross-sectional area provides a form of plastic deformation where the necking occurs. The fractured facets are examined in detail to determine the closely spaced mud structure, where the dry mud solution covers the dust particle surfaces. Because mud liquid, when dried, causes volume shrinkage due to the evaporation of water, high strains are formed in the region where the mud solution dried. Consequently, a rather closely packed mixture of a dry mud solution and fine size dust particles is observed in certain regions, which are marked as “region A” in Fig. 12a. This phenomenon is also seen in Fig. 12b. However, in some other regions (marked as “region B” in Fig. 12a,b), the dust particles are large and are not covered completely by the mud solution because of voids formed in between the large dust particles. In this case, the mud solution flows toward the glass surface due to gravity, and the amount of mud solution present around the large dust particles remains low. Consequently, facets appear to have a porous-like texture (Fig. 12a). Moreover, the coverage area of the porous-like fractured surface over the total area of the fractured surface is estimated from the 3-dimensional images of the fractured surface (as shown in Fig. 12b). The porous-like structures appear as blue lines with a negative textured height (Fig. 12b), and the closely spaced dense layer appears in a brownish/reddish color with a positive texture height (Fig. 12b). The area ratio of the porous-like face to the total area of the face after the tensile test is approximately 78%. This indicates that a closely spaced and dry-mud-solution-covered face surface is typically 22% of the total surface area of the dry mud pellet. The binding force in the closely spaced and mud-solution-covered region is mainly a combination of cohesive and adhesive forces. However, the porous-like textured region of the fractured surface is assumed to be dominated by the adhesion force. Based on this consideration, the ratio of the adhesion force to the combination of the adhesion and cohesion forces is on the order of 3.55, which indicates that the binding force in the dry mud pellet is mainly dominated by the adhesion force. The total work performed during a tensile test is determined by integrating the pulling curve along the displacement, which corresponds to the area under the curve in Fig. 13. Conversely, the tangential force is measured using a micro/nano tribometer; hence, the cohesion force can be determined from the tensile data shown in Fig. 13. In this context, the contributions of the cohesion and adhesion forces to the binding work, which is required to separate the dry mud pellet, are determined using the data in Table 5. In this case, the total work due to adhesion and cohesion is on the order of 1.408 mJ, which corresponds to the area under the curve in Fig. 13. Because the adhesion work covers 78% of the total area and the adhesion work estimated from the tangential force is on the order of 0.119 mJ, the cohesion work due to the dry mud solution around the small dust particles becomes 1.289 mJ. Although the percentage of the cohesion force based on the coverage area obtained from Fig. 12 is small, its effect on the overall binding work is significant, i.e. the ratio of cohesion work, which obtained from tangential force, over the total cohesion and adhesion work obtained from tensile tests is in the order of 90%. Therefore, the formation of a film from the dry mud solution at the interface of the dry mud and the solid surface plays a major role in the removal of the dry mud from surfaces.

## Conclusion

Glasses are used as protective cover for PV panels to protect the photo-active surfaces from environmental effects, such as dust and humidity. Environmental dust particles settle on surfaces and form mud in humid air conditions because of the condensation of water vapor on the dust particles. Because dust particles contain alkaline and alkaline earth metal compounds, they dissolve in water and form a mud solution, which flows in between the dust particles as a result of gravity and sediment at the solid surfaces. Therefore, a thin mud solution film forms in between the dry mud and the solid surface once the mud dries. This film alters the characteristics of the solid surface and the binding forces at the dry mud-solid interface. The present study examined the chemo-mechanical characteristics of the dry mud formed from environmental dust particles are examined in relation to dry mud adhesion to glass surfaces. The dust particles, mud solution, and dry mud were analyzed using analytical tools including scanning electron and atomic force microscopy, energy dispersive spectroscopy, X-ray diffraction, and plasma mass spectroscopy. The friction coefficient of the glass surface after dry mud removal was measured, and the adhesion and cohesion work required to remove the dry mud from the glass surface was determined using tangential force measurements and tensile test data. The results indicated that dust particles have various sizes and that small dust particles attach to large particle surfaces due to the electrostatic charges. Alkaline and alkaline earth metals dissolve in water, creating a mud solution that is basic in character (pH = 7.5). The mud solution forms crystals on the solid surface upon drying. Some dry mud residue is left on the glass surface after cleaning with a pressurized distilled water jet. The presence of dry mud residue was associated with a strong adhesion of the dry mud occurring locally on the glass surface. The dried liquid film formed by the mud solution at the interface of the dry mud and glass surface is responsible for the improved adhesion. The measured adhesion work due to the tangential force component during the mechanical removal of the dry mud from the glass surface demonstrates that the adhesion work is significantly greater than the frictional work. The tensile test data demonstrate that the binding forces for the dry mud consist of cohesion and adhesion forces. By considering the area ratio of the closely spaced small dust particles covered by the dry mud solution on the fractured surface to the total area of fractured test sample surface, the cohesion force acts on 22% of the fractured pellet surface. Although the coverage of the fractured area is small in terms of the cohesive forces, the cohesion work associated with binding the dry mud is greater than the adhesion work.

## Additional Information

**How to cite this article**: Hassan, G. *et al*. Chemo-Mechanical Characteristics of Mud Formed from Environmental Dust Particles in Humid Ambient Air. *Sci. Rep.* **6**, 30253; doi: 10.1038/srep30253 (2016).

## Figures and Tables

**Table 1:** Elemental composition of the dust particles (wt%).

	**Si**	**Ca**	**Na**	**S**	**Mg**	**K**	**Fe**	**Cl**	**O**
Size > 2 μm	11.1	7.1	3.2	2.1	2.4	1.2	1.2	1.1	Balance
Size < 2 μm	11.7	7.9	4.9	1.8	3.7	2.5	1.1	2.4	Balance

**Table 2:** Inductively coupled plasma spectroscopy (ICP) data (ppb) for the mud solution after the dust particles were dissoluted in desalinated water for 8 h.

**Ca**	**Na**	**Mg**	**K**	**Fe**	**Cl**
309800	44600	69950	33400	1830	37600

**Table 3:** Elemental composition of the crystals formed after drying the mud solution on a glass surface (wt%).

	**Si**	**Ca**	**Na**	**S**	**Mg**	**K**	**Fe**	**Cl**	**O**
40 °C	0.6	20	1.4	9.1	0.4	0.4	0.6	1.7	Balance

**Table 4:** Elemental composition of the dry mud residues on a glass surface (wt%).

	**Si**	**Ca**	**Na**	**S**	**Mg**	**K**	**Fe**	**Cl**	**O**
40 °C	1.6	24	1.2	2.2	0.4	0.5	0.8	0.9	Balance

**Table 5:** Adhesion work obtained from the tangential force measurements.

	**Adhesion Work (mJ)**
Dry Mud	0.119 ± 0.008
Mud Solution	0.536.1 ± 0.01
